# Optimizing palliative chemotherapy for advanced invasive mucinous adenocarcinoma of the lung

**DOI:** 10.1186/s12885-021-08472-6

**Published:** 2021-06-26

**Authors:** Yoon Jung Jang, Dong-gon Hyun, Chang-Min Choi, Dae Ho Lee, Sang-We Kim, Shinkyo Yoon, Woo Sung Kim, Wonjun Ji, Jae Cheol Lee

**Affiliations:** 1 Department of Oncology, Asan Medical Center, University of Ulsan College of Medicine, 88, Olympic-Ro 43-Gil, Songpa-Gu, Seoul, 05505 Republic of Korea; 2 Department of Pulmonary and Critical Care Medicine, Asan Medical Center, University of Ulsan College of Medicine, 88, Olympic-Ro 43-Gil, Songpa-Gu, Seoul, 05505 Republic of Korea

**Keywords:** Adenocarcinoma of the lung, Mucinous, Stage IV, Prognosis, Treatment outcome

## Abstract

**Background:**

A primary pulmonary invasive mucinous adenocarcinoma (IMA) is a rare subtype of invasive adenocarcinoma of the lung. The prognosis of advanced IMA depending on chemotherapy regimen has not been fully investigated. Here, we compared the clinical outcomes of patients with advanced IMA treated with different palliative chemotherapies that included novel therapeutics.

**Methods:**

This single-center retrospective study included a total of 79 patients diagnosed with IMA and treated with palliative chemotherapy. The primary outcome was the comparison of overall survival according to palliative chemotherapy type. Risk factors associated with death were evaluated as a secondary outcome.

**Results:**

The study cohort of 79 patients comprised 27 progressive or recurrent cases and 52 initial metastatic patients. Thirteen patients (16.5%) received targeted therapy and 18 cases (22.8%) received immunotherapy. When we compared the survival outcomes of the different treatment regimens, patients with IMA treated by immunotherapy (undefined vs. non-immunotherapy 17.0 months, *p* < 0.001) had better overall survival rates. However, there was no difference in the prognosis between the cases treated with a targeted therapy (35.6 vs. non-targeted therapy 17.0 months, *p* = 0.211). None of the conventional regimens produced a better outcome. By multivariable analysis, immunotherapy (HR 0.28; 95% CI 0.11–0.74; *P* = 0.008) was found to be an independent prognostic factor for death.

**Conclusions:**

This study suggests that immunotherapy for patients with advanced IMA may provide favorable outcomes than other chemotherapy options.

## Background

A primary pulmonary invasive mucinous adenocarcinoma (IMA) is an adenocarcinoma variant composed of histologically goblet and/or columnar cells with abundant intracytoplasmic mucin in the lung tumor [1]. In the 2015 World Health Organization (WHO) classification, IMA was reclassified into one of the various subtypes of invasive adenocarcinoma because its clinical, radiologic, and genetic characteristics were found to be distinct from non-mucinous adenocarcinoma [2, 3]. IMA comprises approximately 5% of all non-small cell lung cancers (NSCLCs) [1]. Numerous novel therapies that target oncogene mutations have improved the prognosis for NSCLC patients [4]. However, most advanced IMAs are usually ineligible for clinical trials of tyrosine kinase inhibitors (TKIs) that target NSCLCs because they principally harbor KRAS mutations and rarely contain targetable mutations in IMA such as those in the epidermal growth factor receptor (EGFR) [5, 6]. Almost all patients with advanced IMA to date have received conventional chemotherapy (CTx) [7].

There have been few studies of the clinical outcomes in patients with IMA after the 2015 WHO classification was adopted. Survival outcomes for IMAs are similar to those for non-mucinous adenocarcinomas [8]. Patients with IMA lesions showing acinar-predominant features on computed tomography (CT) exhibit a poorer prognosis [9]. Some studies have suggested that patients with a surgically resected IMA have similar overall survival (OS) outcomes to non-mucinous adenocarcinoma cases [10, 11]. In patients with metastatic IMA, there was no difference found in a previous report between IMA and non-mucinous adenocarcinoma patients [7]. However, the prognosis of IMA in accordance with the treatment used is not as well characterized as that of non-mucinous adenocarcinoma.

Novel therapies have improved the survival results for metastatic lung cancer patients [12]. One of these new treatments involves the use of immune checkpoint inhibitors and several different studies have demonstrated their benefits in metastatic NSCLCs [13–15]. In addition, the use of TKIs for newly characterized oncogenic mutations such as ROS1, anaplastic lymphoma receptor tyrosine kinase (ALK), and BRAF has led to prolonged survival in NSCLC patients [16–18]. However, the response of novel treatments to patients with IMA remains unclear. Our present study thus aimed to further investigate the prognosis of patients with advanced IMA treated with different palliative chemotherapies that included novel drugs.

## Materials and methods

### Study population

This retrospective study included patients with IMA who were treated with palliative chemotherapy between January 2010 and April 2020 at Asan Medical Center. The diagnosis of IMA was based on the histopathologic report from biopsy specimens and in accordance with the WHO classification of lung tumors [19]. The inclusion criteria for the patient subjects were as follows; 1) a pathological diagnosis of advanced IMA which included local recurrence or progressive lesion after surgical resection and initially metastatic disease; 2) the receipt of palliative chemotherapy. A histopathologic finding of mucinous adenocarcinoma for the patients diagnosed prior to the introduction of the 2015 WHO classification was regarded as IMA. Because the aim of the study was to evaluate patients with advanced IMA treated with palliative chemotherapy, patients who received adjuvant chemotherapy after surgical resection at the time of initial diagnosis were also included. Patients were excluded if they had a colloid adenocarcinoma described in their histopathologic report, failed to receive palliative chemotherapy after the diagnosis of advanced IMA.

### Clinicopathological features of the study patients

Patient data including clinical characteristics, tumor mutation status, and treatment options were collected from their medical records. The following clinical variables collected for analysis: age, sex, eastern cooperative oncology group performance status (ECOG PS), initial clinical stage by TNM 8th edition [20], smoking history (never smokers, former smokers, or current smokers), initial chest CT findings (solid mass or consolidation to the dominant presentation), recurred or metastatic sites, mutational status (EGFR mutation, ALK rearrangement, ROS1 rearrangement, or KRAS mutation), and Programmed death (PD)-1 expression. EGFR and KRAS mutations were detected by PCR from paraffin-embedded tumor samples. Fluorescent in situ hybridization was used to identify ALK and ROS rearrangements [21]. Positive for PD-L1 expression was defined by ≥1% of tumor cells.

### Terms of chemotherapy

We defined the term of a chemotherapy line as a period of the same chemotherapy repeated periodically and the term of a chemotherapy cycle as a single dose of chemotherapy treatment on a regular schedule. The type of chemotherapy was classified into three types; conventional chemotherapy as a cytotoxic chemotherapy that is widely used to lung cancer; targeted therapy as a drug to target specific genes and proteins that are involved in the survival of cancer cells; immunotherapy as a substance to stimulate or suppress the immune system to help the body fight cancer. Treatment methods including chemotherapy lines, cycles, conventional chemotherapy regimen, targeted therapy, and immunotherapy were assessed from the start of palliative chemotherapy to the inclusion period.

### Statistical analysis

The data collected for the study patients were compared using the χ^2^ test or Fisher’s exact test for categorical variables, and independent two sample t-test for continuous variables. Progression-free survival (PFS) was defined as the duration from the date of IMA diagnosis until the date of local progression or the development of a new lesion or death or last follow-up. The OS was calculated from the date of IMA diagnosis until the date of death due to any cause. The primary outcome of this study was the comparison of the PFS and OS outcomes in the patients in accordance with the palliative chemotherapy they received. The secondary outcome included the risk factors associated with death. Kaplan–Meier analysis was used to estimate the PFS and OS, whereas a log-rank test was used to test the significance of the differences in these variables. Univariable and multivariable Cox proportional hazards regression models were used to identify risk factors associated with death. A final model was constructed using a stepwise method with backward selection. *P* values < 0.15 in the univariate analysis were set for the entry of variables. Two-sided *p* values < 0.05 were considered to indicate significance. All analyses were performed using SPSS ver. 24.0 (IBM Corporation, Armonk, NY) software.

## Results

### Patient characteristics

A total 79 patients with IMA who satisfied the inclusion criteria comprised 27 cases that underwent palliative chemotherapy for recurrent or progressive IMA lesions that arose after surgery and 52 patients who were diagnosed with an initially metastatic IMA. The characteristics of patients with IMA included in this study are listed in Table 1.

**Table 1:** Clinical and molecular characteristics of the patients with IMA

Characteristics	All (*n* = 79)
Age, median [interquartile range]	62.0 [54.0–72.0]
Female, n (%)	42 (53.2)
ECOG PS, n (%) (*n* = 61)	
0	2 (3.3)
1	59 (96.7)
Smoking history, n (%)	
Never	41 (51.9)
Former	36 (45.6)
Current	2 (2.5)
Initial Chest CT findings, n (%)	
Solid mass	39 (49.4)
Consolidation	40 (50.6)
Initial Clinical Stage, n (%)	
I	3 (3.8)
II	11 (13.9)
III	14 (17.7)
IV	51 (64.6)
M stage, n (%) (*n* = 77)	
M1a	58 (75.3)
M1b	8 (10.4)
M1c	11 (14.3)
Mutational status, n (%)	
EGFR mutation (*n* = 79)	8 (10.1)
ALK rearrangement (*n* = 69)	4 (5.8)
ROS1 rearrangement (*n* = 2)	0 (0.0)
KRAS mutation (*n* = 12)	11 (91.7)
PD-1 expression (*n* = 33)	14 (42.4)

Results are reported as a number (%) or median [interquartile range]. *ECOG* Eastern Cooperative Oncology Group, *PS* performance status, *CT* computed tomography, *EGFR* epidermal growth factor receptor, *ALK* anaplastic lymphoma receptor tyrosine kinase, *PD-1* programmed cell death protein 1

The median age of this cohort was 62.0 years (interquartile range [IQR], 54.0–72.0 years) and there were about an equal number of men and women. All of ECOG PS were either 0 (3.3%) or 1 (96.7%) in patients with records. Most patients were former (45.6%) or never (51.9%) smokers. There was no difference between the two initial chest CT findings. Almost all of the patients (97.5%) included in this study had a distant metastasis; the two patients who did not had received palliative chemotherapy for a local recurrence. The most common M stage was M1a (75.3%), followed by M1c (14.3%). 10.1% (8/79) of the patients were positive for EGFR mutations, and 5.8% (4/69) and 91.7% (11/12) had an ALK rearrangement and KRAS mutation, respectively. None of the study patients was positive for a ROS1 rearrangement. PD-1 was expressed in 42.4% (14/33) of the cohort.

### Palliative chemotherapy

The mean of chemotherapy line was 2.4 (SD, ± 1.1). Of the 79 patients in the study cohort, 19 (24.1%) underwent only first line chemotherapy whereas 36 received 3rd or 4th line chemotherapy (45.6%; Table 2). The median number of chemotherapy cycles was 4.0 (IQR, 2.0–10.0) at the 1st line, 4.0 (IQR, 2.0–8.0) at the 2nd line, and 4.0 (IQR, 2.0–7.5) at the 3rd line of treatment.

**Table 2:** Palliative chemotherapies and therapeutic regimens used in the study population

Characteristics	All (*n* = 79)
Chemotherapy line, n (%)	
First line	19 (24.1)
Second line	24 (30.4)
Third line	21 (26.6)
Forth line	15 (19.0)
Chemotherapy cycle, median [interquartile range]	
First line	4.0 [2.0–10.0]
Second line	4.0 [2.0–8.0]
Third line	4.0 [2.0–7.5]
Conventional chemotherapy regimen, n (%)	
Platinum & Pemetrexed	40 (50.6)
Platinum & Gemcitabine	30 (38.0)
Platinum & Taxane	5 (6.3)
Pemetrexed	27 (34.2)
Gencitabine	9 (11.4)
Taxane	14 (17.7)
Navelbine	12 (15.2)
Others^a^	7 (8.9)
Targeted therapy, n (%)	13 (16.5)
EGFR-TKI	8 (10.1)
ALK-TKI	4 (5.1)
Other TKI^b^	1 (1.3)
Immunotherapy, n (%)	18 (22.8)
Nivolumab	7 (8.9)
Pembrolizumab	3 (3.8)
Atezolizumab	4 (5.1)
Duvalumab	2 (2.5)
Avelumab	1 (1.3)
Other immunotherapies^c^	3 (3.8)

Results are reported as a number (%) or median [interquartile range]. *EGFR* epidermal growth factor receptor, *TKI* tyrosine kinase inhibitor, *ALK* anaplastic lymphoma receptor tyrosine kinase. ^a^Others comprised 3 irinotecan with platinum, 1 navelbine with platinum, 1 gemcitabine with navelbine, and 2 carboplatin treatment cases. ^b^Other TKI was entrectinib. ^c^Other immunotherapies were PDR001, MK1308, and MEDI5752

**Fig. 1:**
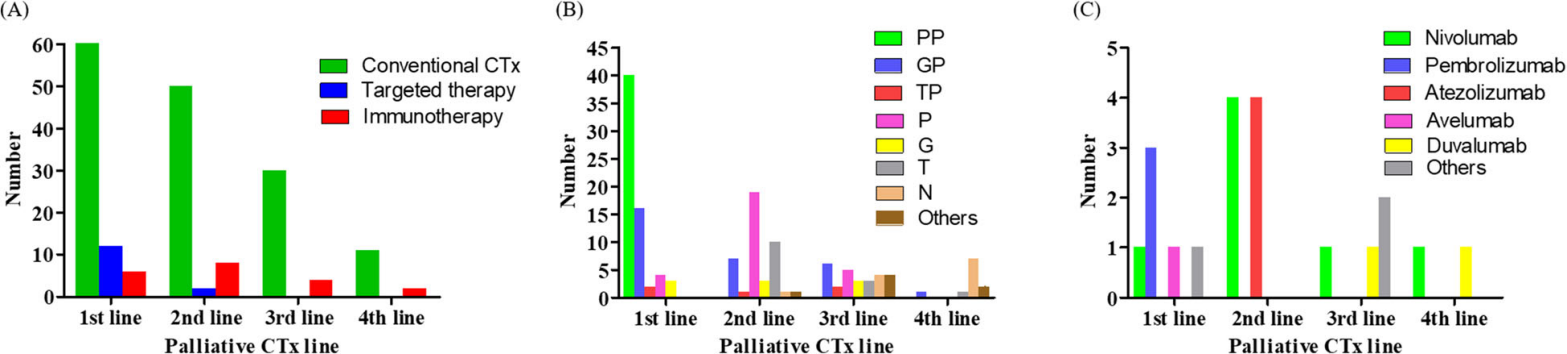
Palliative chemotherapy in accordance with the chemotherapy treatment line. CTx = chemotherapy. PP = platinum with pemetrexed, GP = platinum with gemcitabine, TP = platinum with taxane, P = pemetrexed, G = gemcitabine, T = taxane, N = navelbine. Shown are the numbers of patients stratified by their treatments, i.e. conventional chemotherapy, targeted therapy, and immunotherapy (Panel **A**), conventional regimen (Panel **B**), and the type of immunotherapy (Panel **C**)

The most common agent used for the conventional chemotherapy regimens among the study patients was platinum with pemetrexed (50.6%), followed by platinum with gemcitabine (38.0%). Eight patients (10.1%) received an EGFR TKI and 4 cases (5.1%) were administered an ALK-TKI. One patient (1.3%) received the entrectinib TKI for an NRTK1 fusion. Immunotherapy was administrated to 18 patients (22.8%), of which 4 cases (5.1%) received a combination of immunotherapy and conventional therapy, such as pembrolizumab, pemetrexed, and carboplatin, as a first line. None of the study patients was treated using both immunotherapy and targeted therapy during their course of palliative chemotherapy. Nivolumab (8.9%) was used most commonly, followed by atezolimumab (5.1%). According to the line of chemotherapy, conventional chemotherapy was most common treatment option (Fig. 1A). Targeted therapy was used in the first line in almost all cases (92.3%) whereas only two patients received targeted therapy as a 2nd line regimen, of which 1 received crizotinib after conventional chemotherapy and the other was treated with gefitinib followed by osimertinib. Among the regimens involving conventional agents, platinum with pemetrexed (40/79) was most commonly administrated 1st line therapy, followed by platinum with gemcitabine (16/79) (Fig. 1B). Taxane agents such as docetaxel were usually used as a 2nd line (10/60) whilst navelbine was commonly used in 4th line regimens (7/15). The highest proportion (40.0%) of patients treated by immunotherapy was in the 2nd line, comprising 4 cases receiving nivolumab and 4 receiving atezolizumab (Fig. 1C).

### Progression-free and overall survival

**Fig. 2:**
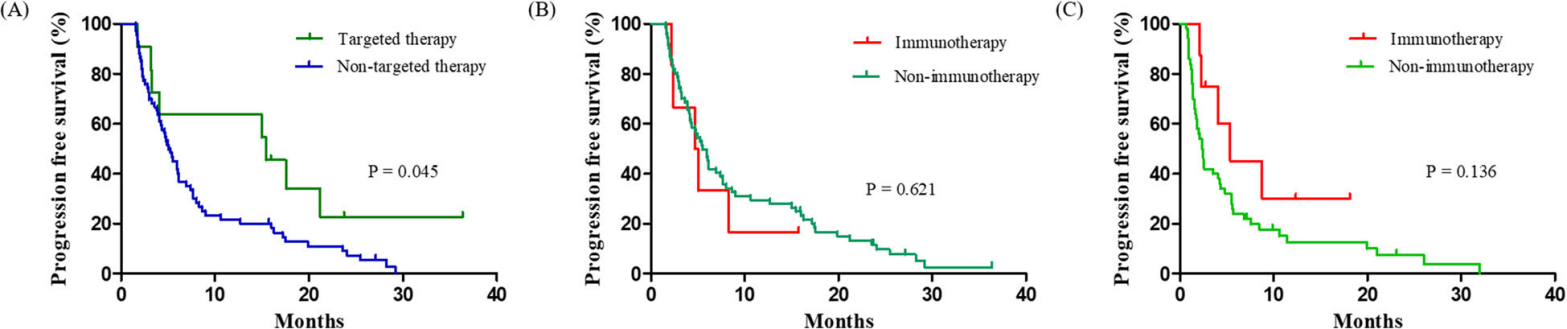
Progression-free survival (PFS) outcomes in the IMA study cohort determined using Kaplan-Meier estimates. The PFS following first line chemotherapy was compared accreting to targeted therapy (Panel **A**), or immunotherapy (Panel **B**). PFS outcome following 2nd line chemotherapy was also analyzed based on immunotherapy (Panel **C**)

**Fig. 3:**
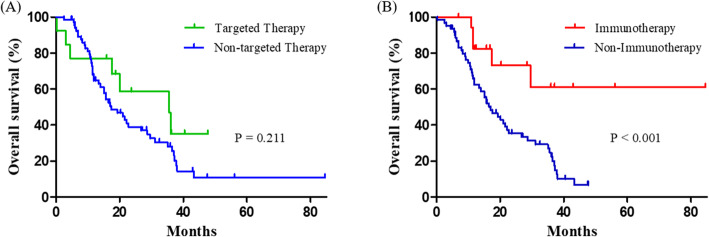
Kaplan-Meier estimates of overall survival (OS) outcomes in the IMA study patients based on targeted therapy (Panel **A**), and immunotherapy (Panel **B**)

The PFS rates were analyzed in our study cohort in accordance with the chemotherapy line (Fig. 2). In the 1st line regimens, patients receiving targeted therapies (median 15.4 months; 95% CI 11.9–19.0) showed a longer PFS (median 5.0 months; 95% CI 4.2–5.9, *P* = 0.045). On the other hand, immunotherapy (median 5.4 months; 95% CI 2.2–8.6) was found to be associated with a trend toward a longer PFS (i.e. not statistically significant (*P* = 0.136) in the 2nd line. The PFS outcomes from targeted therapies in the 2nd line were not analyzed because only two patients were involved. The median survival of the total cohort of 79 patients with IMA was 20.1 months (95% confidence interval (CI), 14.7–25.6). We next compared the OS outcomes in accordance with the therapeutic regimen (Fig. 3). The IMA study patients that received a targeted therapy (median 35.6 months; 95% CI 14.8–56.4) showed a clear non-survival benefit than the patients that underwent non-targeted treatments (median 17.0 months; 95% CI 11.3–23.4, *P* = 0.211). Notably however, patients with IMA treated using immunotherapy (undefined) showed a longer OS than the cases that received non-immunotherapy interventions (median 17.0 months; 95% CI 12.0–22.1, *P* < 0.001). The OS outcomes did not significantly differ among the conventional regimens.

### Prognostic factors

By univariate analysis, M1c (Hazard ratio (HR) 2.37; 95% CI 1.14–4.91; *P* = 0.021) and immunotherapy (HR 0.28; 95% CI 0.11–0.69; *P* = 0.006) were identified as significant variables. Multivariate analysis that controlled for covariables such as the initial metastatic IMA, M stage, pemetrexed and immunotherapy revealed immunotherapy (HR 0.28; 95% CI 0.11–0.74; *P* = 0.008) as an independent prognostic factor for death (Table 3).

**Table 3:** Cox proportional hazards model analysis of death

	Univariable analysis			Multivariable analysis		
Variable	HR	95% CI	*p-*value	HR	95% CI	*p-*value
Initial Stage						
Recurrence Vs. Metastasis	1.59	0.89 to 2.83	.119			
M stage						
M1a Vs. M1b	1.04	0.41 to 2.66	.927			
M1a Vs. M1c	2.37	1.14 to 4.91	.021			
Chemotherapy regimen						
PP	0.79	0.46 to 1.35	.380			
GP	0.80	0.46 to 1.40	.436			
TP	1.86	0.72 to 4.77	.199			
Pemetrexed	1.65	0.97 to 2.82	.068	1.69	0.98 to 2.91	.061
Gemcitabine	1.12	0.55 to 2.30	.756			
Taxane	0.98	0.49 to 1.94	.943			
Navelbine	0.93	0.49 to 1.78	.833			
Other regimens	0.72	0.28 to 1.81	.478			
Targeted Therapy	0.61	0.27 to 1.34	.217			.
EGFR TKI	0.93	0.37 to 2.34	.878			
ALK TKI	0.39	0.10 to 1.63	.198			
Immunotherapy	0.28	0.11 to 0.69	.006	0.28	0.11 to 0.71	.008
Nivolunab	0.34	0.11 to 1.10	.072			
Other immunotherapy	0.04	0.00 to 4.66	.189			

*HR* hazard ratio, *CI* confidence interval, *PP* platinum & pemetrexed, *GP* gemcitabine & platinum, *TP* taxane & platinum

## Discussion

In our current study, we found that the prognosis of patients with IMA who received palliative chemotherapy was variable in accordance with the type of chemotherapeutic intervention. Patients with IMA treated by immunotherapy appeared to have a better outcome than those who received chemotherapy. Of note in particular, immunotherapy was found to be associated with improved survival in multivariable analysis. On the other hand, targeted therapy did not improve the survival outcomes of the advanced IMA cases in our present series, although the PFS was better in these patients compared with those without targeted therapy. In addition, no OS differences were evident based on conventional agents. We thus contend from our current data that immunotherapy should be considered, if possible, as a principal treatment option in patients with advanced IMA.

In our present analyses, the median survival of patients with IMA was 20.1 months. When compared with the findings of previous reports, our measured OS is similar to that reported by Cha et al. of 17.9–20.9 months from patients with metastatic IMA, with no OS differences found between IMA and non-IMA adenocarcinoma cases in that prior study [7]. Several previous clinical trials that compared treatment efficacies reported a median survival of 14.2–21.5 months from the use of conventional chemotherapy [22–24]. On assessing the conventional chemotherapy regimens in our present IMA population, we noted that the combination of pemetrexed or gemcitabine with platinum agents was the most common first line protocol for these treatments. Real-world practices reported in other studies have also shown a similar pattern. In prior reports on non-squamous NSCLC, the most common regimen was carboplatin plus pemetrexed (25.7%) in the US, and pemetrexed-based therapy (68%) in China [25, 26]. Notably, the proportion (about 42%) of patients who received second-line therapies in the American study [25] and the Chinese study [26] was lower than that (75.9%) in our present study. In our current cohort, only 13 patients (16.5%) were found to have oncogenic mutations whereas 11 KRAS mutations were detected in a setting involving 12 patients with IMA in previous reports [27, 28]. Considering that the patients in our current cohort had similar clinicopathologic features and treatment environments to those previously reported for IMA, the clinical outcomes of IMA seems to be comparable to those for non-squamous NSCLC.

It has been well established that KRAS mutations are the most frequent genetic alternations seen in IMA [8]. On the other hand, IMAs usually lack other oncogenic mutations such as EGFR variants, or translocations of ALK or ROS1 [29]. KRAS mutations are mutually exclusive from EGFR mutations or ALK/ROS1 rearrangements based on oncogene addiction theory [30]. The molecular mechanisms driving IMA remain unclear, but some reports have suggested possible mechanisms for identifying novel therapeutic targets. A previous animal study has suggested that the induction of the signature genes FOXA3 or SPDEF, which are enriched in mucin-producing cancers, along with a KRAS mutation in the lung epithelium, is sufficient to develop mucinous lung tumors in transgenic mice [28]. Other studies have revealed that a loss of TTF-1 expression owing to an NKX2–1 mutation, which occurs in approximately 19% of IMA cases, would de-suppress the expression of the mucin-related genes MUC5Ac, MUC5B, and MUC3 [31]. In addition, novel driver mutations, NRG1 fusions, have been recurrently identified in IMA, i.e. as a regulator of goblet-cell formation in human bronchial epithelial cells [32]. The NRG1 protein mediates juxtacrine signals through the HER2:HER3 receptors that may play a role in the transformation and acquisition of goblet-cell morphology in IMA [33]. Recently, several clinical trials have been performed on drugs that target KRAS-mutated IMAs. A phase 1 trial of sotorasib, a small molecule that selectively targets the KRAS G12C subtype, revealed an 88.1% disease control response in NSCLC [34]. Driver mutations such as KRAS thus represent promising future therapeutic targets for the treatment of IMA.

The OS outcomes were found in our present series to be significantly improved in patients treated with immunotherapy. Numerous prior studies have reported that immunotherapy produces favorable outcomes in NSCLCs and that the clinical response predictions in these patients depend on the expression of biomarkers such as PD-L1 or on the tumor mutation burden [35–37]. Previous analysis of the expression of PD-L1 in IMA has indicated that PD-L1 positive tumors are infrequent [38]. However, other findings suggest the possibility of a good response to immunotherapy by IMA as KRAS mutations show an association with this response [30]. Although most prior randomized trials have not been designed to examine treatment differences between molecular subgroups, immunotherapy showed a significantly greater benefit for KRAS mutant tumors in one previous meta-analysis [39]. However, another study using real-world data demonstrated that a KRAS mutation did not confer any significant OS difference [40]. An attractive biological explanation for these discrepant findings is the molecular and environmental diversity of KRAS mutation subgroups [30]. Three of these subgroups have been defined in accordance with the presence of co-mutations, and have differences in terms of the immune environment and the responses to immunotherapy. In addition, IMAs have distinct expression profiles of immune checkpoint regulators such as VTCN1, which represents a potential immunotherapy target [28]. Although the biology to support the benefits of immunotherapy in IMA remains unclear, further studies are warranted to address whether key mutations such as KRAS can offer predictive insights into the immunotherapy responses of IMA tumors.

Our present study had several limitations of note. First, it was a retrospective study with a relatively small sample size from a single institution. However, we tried to include patients with IMA having similar characteristics to minimize possible bias and investigated all of the regimens which patients received as palliative chemotherapy. This is also, to our knowledge, the first study to identify the clinical outcomes of patients with IMA who had received immunotherapy. Prospective, randomized studies will be needed to clarify the clinical outcomes of patients with IMA in accordance with their palliative treatments. A second limitation was that we included patients who had been diagnosed prior to the WHO reclassification with mucinous adenocarcinoma, not IMA. Patients with non-IMA could therefore have been included in our cohort. We did however exclude patients with other variants of invasive adenocarcinoma such as colloids who has been diagnosed before 2015, which would have helped to reduce the heterogeneity of our cohort. A third limitation was that targeted therapies did not show survival benefits in our present series, which contrasts with the findings of previous studies [41]. Although we did observe an improved PFS from 1st line targeted therapies among our current patients, these treatments were not found to be associated with the IMA prognosis in our multivariate analysis. The distinct characteristics of EGFR mutations that have been found to have no female predominance and operate in a different tumorigenic pathway in IMA could have impacted on these targeted treatment responses [42]. Finally, PD L-1 expression was not examined in all the patients in our cohort. This marker is used as a predictor of the response to immunotherapy and a future examination of its status in IMA could provide insights into the causality behind the good response of these cancers to immunotherapy.

## Conclusions

This study suggested that the type of palliative chemotherapy is associated with the prognosis of patients with advanced IMA. Patients with IMA treated by immunotherapy may have better outcomes than those treated using other chemotherapies. Further studies are needed to demonstrate the efficacy of these treatments as a palliative intervention for patients with IMA.

## Data Availability

The datasets used and/or analysed during the current study are available from the corresponding author on reasonable request.

## Abbreviations

IMA: Invasive mucinous adenocarcinoma
WHO: World Health Organization
NSCLC: Non-small cell lung cancer
TKI: Tyrosine kinase inhibitor
EGFR: Epidermal growth factor receptor
CTx: Chemotherapy
CT: Computed tomography
OS: Overall survival
ALK: Anaplastic lymphoma receptor tyrosine kinase
PD: Programmed death
PFS: Progression-free survival
CI: Confidence interval
HR: Hazard ratio
